# Post-intensive care unit syndrome: trends and research foci in a bibliometric analysis

**DOI:** 10.1590/1518-8345.8124.4927

**Published:** 2026-10-09

**Authors:** Natália Soares da Silva, Paulo Jorge Pereira Alves, Elsa Maria Oliveira Pinheiro de Melo, Rafael Alves Bernardes

**Affiliations:** 1 Universidade Católica Portuguesa, Faculdade de Ciências da Saúde e Enfermagem, Porto, Portugal.; 2 Universidade de Aveiro, Escola Superior de Saúde, Aveiro, Portugal.; 3 Universidade Católica Portuguesa, Centro de Investigação Interdisciplinar em Saúde, Lisboa, Portugal.; 4 Universidade de Aveiro, Unidade de Investigação Instituto de Biomedicina, Aveiro, Portugal.; 5 Universidade Católica Portuguesa, Faculdade de Ciências da Saúde e Enfermagem, Lisboa, Portugal.

**Keywords:** Bibliometrics, Critical Care, Practice Guideline, Quality of Life, Follow-Up Studies, Critical Care Nursing.

## Abstract

**Objective:**

to identify the main areas of focus and trends in research on Post-Intensive Care Syndrome, with an emphasis on patient follow-up after discharge from the Intensive Care Unit.

**Method:**

search conducted on Web of Science from 2012 to November 2024. The Bibliometrix package and BiblioShiny platform in RStudio were used to explore keywords, trends and co-occurrences, journals, authors, co-citations, and collaboration networks. 120 articles on Post-Intensive Care Syndrome in adults were included.

**Results:**

the main keywords include critical illness, quality-of-life, and posttraumatic stress disorder. The most prolific authors are Needham D.M. and Sevin C.M., and the most relevant journals are Critical Care and BMJ Open. The United States leads in publications, followed by the Netherlands and Japan.

**Conclusion:**

most studies address a single component of the syndrome, and comparisons between studies are hampered by the diversity of assessment tools. Greater international collaboration, long-term studies, and randomized controlled clinical trials are needed.

## Highlights

**(1)** Research on PICS has grown, focusing on rehabilitation and quality of life.

**(2)** Studies address domains of PICS in isolation, limiting integrated approaches.

**(3)** There is a lack of consensus and standardized tools for effective screening and intervention.

## Introduction

Advances in intensive care medicine, namely the implementation of evidence-based therapeutic guidelines and the continuous training of healthcare professionals, have contributed significantly to reducing short-term mortality in critically ill patients^1^ ^),(^ ^2^. However, although hospital survival has improved substantially, the quality of life and long-term functional outcomes of patients remain a major challenge^2^ ^),(^ ^3^ ^),(^ ^4^.

In recent years, healthcare professionals have shown a growing concern for understanding the sequelae of patients after discharge from the Intensive Care Unit (ICU). Post-Intensive Care Syndrome (PICS) was first described by the American Society of Critical Care Medicine (SCCM) at the Global Critical Care Conference in 2010, defining it as a set of physical, cognitive, and psychological deficits that can affect both ICU survivors and their families (Post-Intensive Care Syndrome-Family - PICS-F) ^5^ ^)(^ ^6^.

Given the consequences of the syndrome, it is essential to further study it, understand the risk factors involved, and develop effective rehabilitation strategies to minimize its adverse effects^6^. Continued research on multidisciplinary interventions, including early rehabilitation programs, psychological support, and post-discharge monitoring, can play a key role in improving outcomes for patients and their families^4^.

Post-Intensive Care Syndrome comprises six defining attributes: multidimensional impairments, either new or aggravated; physical dysfunction; psychological distress; cognitive impairment; failed social reconstruction; and impaired symptoms that persist after discharge from the ICU^7^.

It is a complex syndrome involving multiple interacting dimensions that significantly affect survivors’ quality of life and social reintegration^8^ ^)(^ ^9^ ^)(^ ^10^.

PICS has a high prevalence, therefore requiring long-term outpatient follow-up after discharge. Its prevalence varies widely depending on the population studied, the diagnostic criteria used, and the conditions of post-discharge follow-up. Of all people who survive ICU admission, about 50% will have at least one of the problems associated with PICS^11^. In some cases, 64.9% of patients show symptoms at their first consultation after discharge from the ICU^9^.

For PICS assessment, consensus-based recommendations emphasize the evaluation of cognition, mental health, and physical function using various tools^5^ ^),(^ ^8^ ^),(^ ^12^.

Risk factors for PICS are classified into patient history, disease severity, medical care, and environmental stress^13^. Advanced age, female gender, previous mental health problems, severity of illness, negative experience in intensive care admission, and delirium represent significant risk factors for PICS^14^. It should be noted that one of the factors associated with an increased risk of developing cognitive impairment after admission to the ICU included the occurrence of *delirium* ^4^ ^),(^ ^9^ ^),(^ ^15^.

The background history of patients admitted to the ICU is the main contributor to the occurrence of SIRS, whether due to specific treatments or the severity of the critical illness itself^7^.

The experience of family members and/or informal caregivers of critically ill patients can trigger significant psychological symptoms. In this context, it is essential to recognize that the health of these caregivers is a crucial component of the therapeutic process. Family Post-Intensive Care Syndrome (PICS-F) was observed in 19.1% of family members and/or caregivers, with the most frequent change being increased family burden and anxiety^9^. In addition, family members themselves experience periods of continuous stress and anxiety after the family member is discharged^16^.

The prevention of PICS is closely related to the early identification of potential modifiable risk factors. The application of a grouped care plan such as the ABCDEF bundle (analgesia and sedation selection, spontaneous ventilation trials, coordination between spontaneous ventilation trials and awakening, delirium prevention, exercise and early mobilization/rehabilitation, and finally, communication and family involvement) can improve patient-centered care as well as the experience of patients and their families^9^.

A negative patient experience during ICU admission can be modified by implementing strategies to reduce delirium, increase early mobilization, optimize pain control, and reduce or modify the use of physical restraints^17^.

There is no consensus in the literature on the ideal model for follow-up of individuals after critical illness. However, three pillars should be observed: identification of the risk of PICS and its prevention; identification of PICS in its multiple components; and a multidisciplinary approach^18^. 

Given the scarcity of specific literature and the absence of consolidated guidelines on PICS, there is a need to map the depth and nature of investigations into long-term outcomes, the effectiveness of interventions, and methodological standardization. Thus, the objective of this study is to identify the main focuses and trends in research on Post-Intensive Care Syndrome, with an emphasis on the follow-up of patients after discharge from the Intensive Care Unit.

## Method

### Study design

This study applied bibliometric analysis as a research design, which has been recognized as an effective methodology for quantitatively studying scientific production^19^ ^),(^ ^20^. This approach allows the identification of patterns, trends, and the evolution of scientific knowledge over time by systematically analyzing large data sets. The analysis was conducted based on the main laws of bibliometrics: Bradford’s Law, applied to define the core of the most productive journals and categorize the others into zones of dispersion; Lotka’s Law, used to distribute authors according to their productivity, identifying the most active ones in the field; and Zipf’s Law, which guided the refinement and grouping of terms, creating a representative list of the most frequent concepts in the analyzed articles^19^.

For the analysis, the Bibliometrix and BiblioShiny tools in the RStudio software, developed^20^ for descriptive statistics, and the VOSviewer software, which allows the construction and visualization of bibliometric maps^21^ based on keyword co-occurrence networks, collaboration between authors, and citations, were used.

In this context, the following research questions were formulated: What are the most relevant keywords used in research studies related to PICS? What is the trend in research patterns, emerging interests, and changes in practices in different research contexts over the years? What are the main sources of publication of research articles?

### Data screening and analysis

In this bibliometric analysis study, two reviewers independently examined all publications, reading the title and abstract. Discrepancies were resolved by consensus, and in cases where no agreement was reached, a third reviewer acted as the decision maker. The publication collection process is shown in Figure 1. The search was conducted on Web of Science, as in other studies^22^ ^),(^ ^23^, from November 20, 2024; thus, data for the year 2024 are partial. This database provides researchers with a diverse range of high-quality publications^24^, with standardized bibliographic records and temporal consistency, ensuring greater rigor, transparency, and reproducibility in bibliometric analysis. It is recognized, however, that the choice of a single database may limit the representativeness of the results.

**Figure 1:**
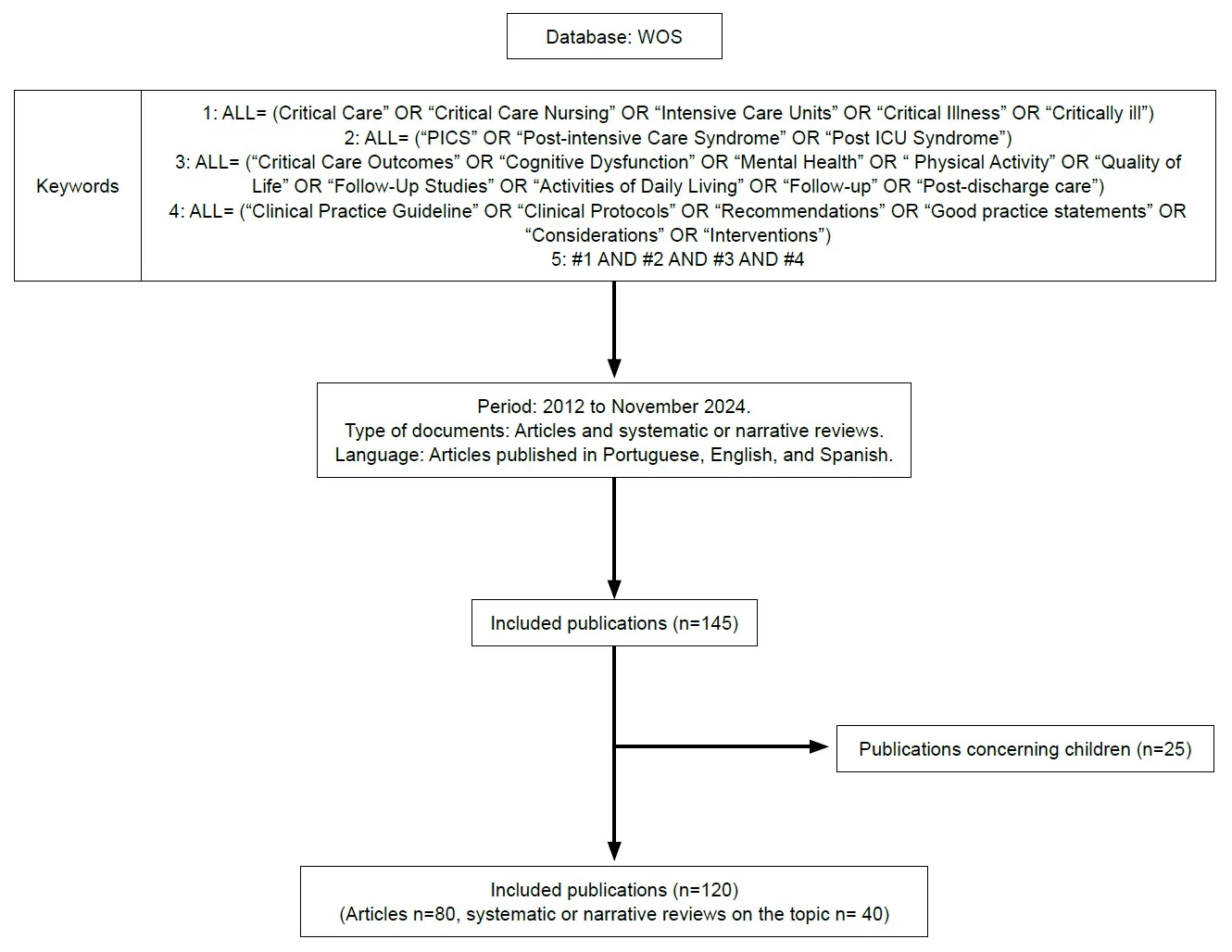
Flowchart of the bibliometric review. Aveiro, Portugal, 2024

The inclusion criteria were: (i) studies published since 2012, as this was when the first formal definition of this syndrome appeared^4^; (ii) articles published in Portuguese, English, and Spanish; (iii) studies focusing on PICS and/or PICS-F; (iv) research focused on the follow-up of patients after discharge from the ICU; (iv) studies with an adult population (>18 years).

The exclusion criteria were: grey literature, book chapters, conference proceedings/abstracts.

After applying the selection criteria, the documents were saved in BibTeX format. The final file was exported to Excel for manual review. 

It is recognized that the search strategy adopted is restrictive and may exclude some relevant studies. In future studies, it can be improved by expanding synonyms or making blocks more flexible.

### Statistical analysis

A co-occurrence analysis was performed in VOSviewer with the 50 main Author Keywords from the 120 articles, selected according to Bradford’s Law zones and previously normalized by removing duplicates and unifying synonyms, based on a systematic manual review of the terms. The network analysis provides several clusters in the network, represented by different colors, with a frequency ≥ 5 in the keyword grouping. The size of the circular node on the map is proportional to the frequency of the keyword, and the distance and thickness of the lines between words indicate the strength of their relationship. While the overall distance between keywords reflects their correlation, generally, a shorter distance between two keywords implies a stronger relationship between them. The color of the node shows the cluster to which the keyword is associated. Each cluster belongs to a research theme represented by the keywords and their links.

For data analysis, the Bibliometrix package, developed in the R programming language, was used within the RStudio environment. This package allowed the extraction and organization of the main research topics, most productive authors, highest impact journals, countries with the greatest scientific contribution, and international collaborations in the field of study. 

In addition, the most relevant articles were identified based on the number of citations, providing a comprehensive view of academic influence in the field of PICS.

The bibliometric analysis followed four elements:

Keyword Co-occurrence Analysis: identification of predominant themes and emerging trends based on keyword frequency and clustering.

Citation Analysis: evaluation of the most cited articles, authors, and journals in the dataset.

Co-authorship Network Analysis: examination of collaborations between authors, institutions, and countries.

Co-citation Analysis: mapping intellectual structures through the analysis of frequently co-cited works.

## Results

Studies related to PICS began to formally emerge in 2012, and since then, scientific production has shown continuous growth over the years.

### Keywords

The analysis of keywords and their trends over time allows us to identify research patterns, emerging interests, and changes in practices in different research contexts.

From 2015 onwards, a critical mass of existing articles allowed the identification of keywords with minimum frequency. The prominence of specific keywords became evident in 2021, with terms such as “posttraumatic stress disorder,” “quality of life,” and “critical illness.” Recently, the most frequently used terms are “impact,” “unit,” and “rehabilitation,” reflecting an approach centered on post-discharge outcomes with a focus on the recovery of critical illness survivors.

Figure 2 shows the most searched terms, as well as their co-occurrences, with a frequency ≥ 5 co-occurrences, providing a greater understanding of the research landscape.

The network map illustrates the highly interconnected and multidisciplinary structure of research focused on post-intensive care syndrome, which emerges as a central element of the network, articulating with the main themes in the literature. Among these, critical illness stands out as the most robust focus in the field, with 96 occurrences and a total connection strength of 813. 

Other thematic clusters (mental health, delirium/clinical management, rehabilitation, and cognitive function) are part of this network, demonstrating the complexity of the field of study. The red cluster focuses on the emotional repercussions and psychosocial determinants associated with PICS. The studies gathered here emphasize the high burden of psychological morbidity. The relevance of the social context is evidenced by the presence of the term “caregivers”, which appears five times, with a total connection strength of 41. This cluster has a strong synergy with the purple cluster, underlining the importance of integrating family support and caregiver dynamics into follow-up approaches. 

**Figure 2:**
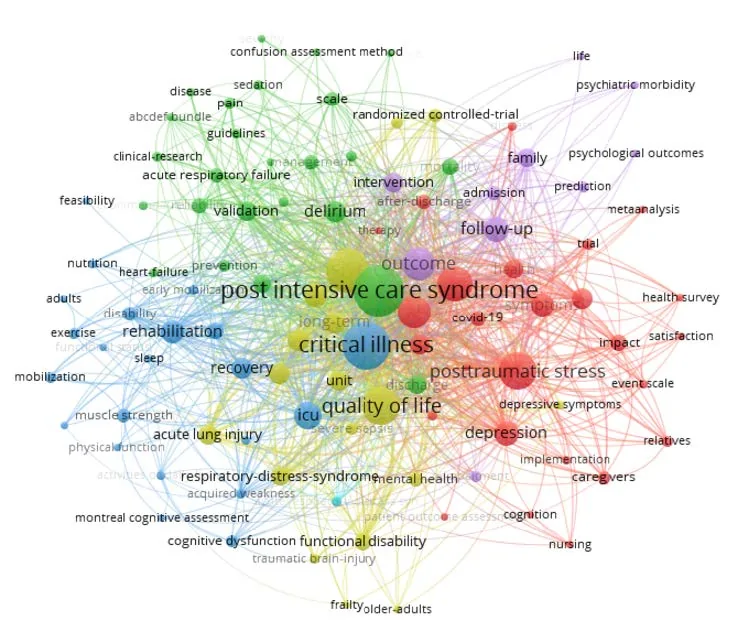
Co-occurrence analysis of search terms. Aveiro, Portugal, 2024

The green cluster brings together themes related to the acute phase of critical illness. “Delirium” stands out as the most prominent clinical topic, with 21 occurrences and a total link strength of 175, reflecting its central role as a marker of severity, frequent complication, and predictor of physical, cognitive, and psychological outcomes in the post-discharge period.

The blue cluster focuses on the physical dimensions of recovery. The concepts of rehabilitation and recovery structure this group, demonstrating the growing attention given to physical rehabilitation interventions and functional recovery after a stay in the ICU.

Finally, the yellow cluster covers the persistent cognitive effects after critical illness, including deficits in memory, attention, and executive functions. Quality of life emerges as a central node, linking cognitive and functional sequelae to the overall outcomes of survivors.

The groups of terms indicate a transition from diagnostic research to integrated and personalized management, with an increasing focus on biological mechanisms and the patient experience.

### Journals

The number of articles published in specialized journals is an indicator of the relevance of the topic within the scientific community. Table 1 shows the journals that published the most articles in the field of PICS, according to the h-index and the total number of citations.

The journal with the most publications and greatest relevance is Critical Care, which published a total of 10 papers between 2016 and 2024, with 543 citations, and has an h-index of 7 and a g-index of 10.

This list includes eight journals with their first publication on the subject under analysis from 2020 onwards, highlighting the growing consolidation of the topic as a relevant field of research.

**Table 1:** Analysis of publications: h_index and citations. Aveiro, Portugal, 2024

Journals	h_index*	g_index^†^	*TC* ^*‡*^	*NP* ^*§*^	*PY-start* ^*||*^
Critical Care	7	10	543	10	2016
BMJ Open	6	8	201	8	2017
Critical Care Medicine	5	8	800	8	2014
Annals Of The American Thoracic Society	3	4	160	4	2016
Critical Care Explorations	3	4	36	4	2022
Current Opinion In Critical Care	3	5	79	5	2012
Intensive And Critical Care Nursing	3	4	32	4	2020
Journal Of Intensive Care	3	3	52	3	2023
Acute Medicine & Surgery	2	2	8	2	2024
Australian Critical Care	2	3	24	3	2022
Critical Care Nurse	2	2	8	2	2022
Journal Of Advanced Nursing	2	2	8	2	2021
Journal Of Clinical Medicine	2	3	15	3	2021
Journal Of Critical Care	2	3	184	3	2015
*Medicina Intensiva*	2	2	17	2	2019

*h-index = Metric that provides a balanced view of author productivity and impact; ^†^g-index = Index that amplifies citations of the most cited articles to highlight distinctions between researchers; ^‡^TC = Total citations; ^§^NP = Number of publications; ^||^PY-start = First publication

In addition, it is important to note that this list includes three nursing journals, Intensive And Critical Care Nursing, Critical Care Nurse, and Journal of Advanced Nursing, which provides information on the impact and consistency of these journals’ publications in the field of nursing and intensive care.

### Analysis of authors and citations

Table 2 summarizes the authors’ contributions to PICS research.

Evaluated in terms of the number of articles published, h-index, g-index, and total number of citations obtained, Needham stands out with 12 publications and the highest number of citations: 808. Together with Hopkins, they are the authors who have been publishing on the subject for the longest time.

With more recent publications, since 2018, Sevin stands out with nine publications and 501 citations, that is, the impact of his publications is well represented by the number of citations of his most cited articles, and Jackson, who has 7 articles with a total of 765 citations and an h-index of 6. This author has a solid base of well-cited works with significant impact.

**Table 2:** Citation analysis - author evaluation. Aveiro, Portugal, 2024

Authors		h_index*	g_index^†^	*TC* ^*‡*^	NP^§^	PY_start^||^
Needham D.M.		8	12	808	12	2014
Sevin C.M.		7	9	501	9	2018
Hopkins R.O.		6	7	668	7	2012
Jackson J.C.		6	7	765	7	2018
Akhlaghi N.		4	4	150	4	2019
Bose S.		4	4	150	4	2019
Brown S.M.		4	4	150	4	2019
Dinglas V.D.		4	5	150	5	2019
Ely E.W.		4	4	434	4	2018

*h-index = Metric that provides a balanced view of author productivity and impact; ^†^g-index = Index that amplifies citations of the most cited articles to highlight distinctions between researchers; ^‡^TC = Total citations; ^§^NP = Number of publications; ^||^PY_start = First publication

Figure 3 presents the list of the most cited articles, identifying the authors, the year of publication, the title of the article, and the number of citations. 

Among the most cited articles are “Co-occurrence of post-intensive care syndrome problems among 406 survivors of critical illness” and “Anxiety, depression, and post-traumatic stress disorder after critical illness: a prospective UK-wide cohort study,” both from 2018.

The article^25^ “Exploring the Scope of Post-Intensive Care Syndrome Therapy and Care: Engagement of Non-Critical Care Providers and Survivors in a Second Stakeholders Meeting,” with 281 citations, contributed to raising awareness and education about PICU.

**Figure 3:** Citation analysis. Aveiro, Portugal, 2024

Author, year	Title	Citations
Marra, et al., 2018	Co-occurrence of Post-Intensive Care Syndrome Problems Among 406 Survivors of Critical Illness	287
Elliott, et al., 2014	Exploring the Scope of Post-Intensive Care Syndrome Therapy and Care: Engagement of Non-Critical Care Providers and Survivors in a Second Stakeholders Meeting	281
Hatch, et al., 2018	Anxiety, Depression and Post Traumatic Stress Disorder after critical illness: a UK wide prospective cohort study	281
Mikkelsen, et al., 2020	Society of Critical Care Medicine’s International Consensus Conference on Prediction and Identification of Long-Term Impairments After Critical Illness	202
Fuke, et al., 2018	Early rehabilitation to prevent postintensive care syndrome in patients with critical illness: a systematic review and meta-analysis	130
Sevin, et al., 2018	Comprehensive Care of ICU Survivors: Development and Implementation of an ICU Recovery Center	126
Brown, et al., 2019	Approaches to Addressing Post-Intensive Care Syndrome among Intensive Care Unit Survivor	126
Gayat, et al., 2018	Determinants of long-term outcome in ICU survivors: results from the FROG-ICU study	124
Schofield-Robinson, et al., 2018	Follow-up services for improving long-term outcomes in intensive care unit (ICU) survivors (Review)	121
Jensen, et al., 2015	Impact of follow-up consultations for ICU survivors on post-ICU syndrome: a systematic review and meta-analysis	110
Major, et al., 2016	Surviving critical illness: what is next? An expert consensus statement on physical rehabilitation after hospital discharge	80

With over 100 citations and published in 2019, the article^26^ “Approaches to Addressing Post-Intensive Care Syndrome among Intensive Care Unit Survivors” is also noteworthy.

### Distribution of scientific output

The distribution of scientific output on PICS, considering both international collaborations and national publications, revealed that the United States leads significantly in both the absolute number of publications and international collaborations, which highlights its centrality in research on this topic.

The Netherlands and Japan come next, demonstrating strong involvement in scientific production and standing out for their active global collaboration network. China, despite having a considerable volume of publications, has not established significant international collaborations within the sample analyzed. 

The results suggest that scientific production on PICS is concentrated in certain academic centers, driven by countries that demonstrate greater investment in research on post-ICU care, including rehabilitation.

## Discussion

Although the bibliometric analysis was only conducted on Web of Science, it allowed us to answer the research questions and provide a comprehensive overview of the evolution of research related to PICS, as well as to verify a growing trend in original research articles and reviews on PICS. However, this limitation, as well as the small number of articles, may introduce selection bias, reducing the generalizability of the results.

The analysis of the most frequently used terms in the research provides valuable insights into the research landscape^20^ ^),(^ ^27^, with the emergence of terms such as “impact” and “rehabilitation.” These terms reflect the transition from initial research, which emphasized the characterization of the syndrome, to a more applied approach focused on rehabilitation and quality of life, which is significantly affected after admission to the ICU^8^ ^),(^ ^19^. The elements of the clusters confirm the multidimensional complexity of Post-Intensive Care Syndrome, broaden understanding with new dimensions and technologies, and question the model of follow-up after admission to Intensive Care and methodological heterogeneity.

The evolution of research over the years has shown an increase in interest in topics such as post-traumatic stress disorder, quality of life, and the impact of critical illness, highlighting the need for holistic approaches and multidisciplinary interventions that facilitate the patient’s adaptation to their new health situation. Descriptive knowledge has matured, but interventional, predictive, and translational research remains underdeveloped.

Bibliometrics revealed that research on PICS is concentrated in scientific journals of great relevance in the field of critical care and intensive medicine, with emphasis on the journals: Critical Care^22^, BMJ Open, and Critical Care Medicine^23^, which had the highest productivity and citation rates. The presence of three nursing-specific journals in the list of the most relevant demonstrates the growing importance that nurses attach to the detection, management, and rehabilitation of ICU survivors.

Nurses play a key role in minimizing PICS through interventions focused on early mobilization, delirium management, family support, and discharge planning^28^. In the follow-up of survivors and families, nursing interventions also aim to activate effective coping strategies, promote self-care, restore patient autonomy, and prevent caregiver fatigue^29^. The follow-up of patients after ICU admission represents an emerging area in healthcare, aiming to identify and address the long-term consequences of ICU stay more effectively than standard care^30^. However, the available data are limited in terms of effective interventions to be implemented and highlight the lack of standardization of a model for following up patients after critical illness, with gaps in the implementation of integrated and longitudinal approaches.

A high prevalence of cognitive impairment, anxiety, depression, physical weakness, lifestyle changes, and medication-related problems has been identified, justifying intervention in patients after intensive care admission^31^. ICU-related stress is associated with a decrease in patients’ quality of life after discharge from the ICU^9^. The concept of the “altered self” is the transformation of the self reflecting the new physical limitations experienced, as well as the way in which the mobilization of the individual’s intrinsic characteristics facilitates adaptation to their new identity^32^.

Monitoring patients can help them create their own narrative of illness and regain a sense of coherence, but randomized clinical trials are needed to standardize interventions and outcome measures^33^.

The authors^5^ recommend systematic assessments and suggest some screening tools to evaluate each component of PICS, highlighting the importance of prioritizing high-risk patients and referring them to more detailed services and/or assessments. Systematic and frequent assessment of PICS is necessary throughout the continuum of care^25^.

It is important to identify risk factors for the development of PICS, and the multidisciplinary team should be aware of modifiable risk factors. Increased understanding of risk factors has improved the ability to identify high-risk patients for screening and intervention^17^. The authors^10^ assessed the incidence of PICS in COVID-19 survivors and identified delirium and tracheostomy as associated factors. The nursing team plays a key role in both the early identification of risk factors and the implementation of bundles, reducing the risk of developing PICS.

It is important to conduct studies that examine the implementation of recommendations, including their feasibility, acceptability, sustainability, and fidelity, in addition to assessing the impact of recommendations on long-term outcomes for survivors^5^. Research should focus on patients and care transitions^26^, with effective care transitions ensuring continuity between treatment in the ICU, on the ward, and ongoing support in the community and at home.

The bibliometric analysis also revealed inequalities in the distribution of scientific production on PICS and related areas between countries and continents; additionally, the linguistic restriction adopted (Portuguese, English, and Spanish) stands out as a relevant methodological aspect of the research. Similar to other studies^22^ ^),(^ ^23^, the United States leads significantly in both the number of publications and international collaborations, which may be related to robust investment in post-ICU care and longitudinal research. The Netherlands and Japan also stand out as active research hubs, reflecting the development of advanced strategies for rehabilitation and restructuring of intensive care.

On the other hand, China, despite having a significant volume of publications, showed low international collaboration, possibly due to language barriers or a research model more focused on the internal clinical reality. Portugal, in turn, shows reduced participation and a lack of international collaborations in the sample analyzed. This can be attributed to funding difficulties and the lack of collaborative networks with international intensive care groups, particularly in Europe.

PICS remains a growing concern, and there is consensus in the literature on the need to improve mechanisms for early identification and follow-up of survivors of critical illness. Systematic and frequent assessments of the syndrome should be carried out throughout the continuum of care, giving priority to high-risk patients for early intervention^5^, and investigating their molecular biology or genetic predispositions^22^.

Recent studies point to the need for effective transition strategies, ensuring continuity of care between discharge from the ICU and return to daily life^26^. Nurses are crucial as coordinators of this follow-up due to their experience in managing complex care and their ability to liaise between the patient, family, and multidisciplinary team^29^.

The growing attention given to PICS highlights the importance of expanding international collaborations, accelerating research in related areas, promoting multicenter research networks, and ensuring that new scientific evidence is applicable to diverse clinical settings. Strengthening these networks can contribute to a more equitable and effective approach to follow-up after ICU admission, benefiting not only patients but also their families and the professionals involved in their recovery/rehabilitation.

Research on PICS has limitations, focusing only on short-term clinical effects, with little long-term analysis and multinational studies.

In addition, many studies analyze only one dimension of the syndrome, without exploring the interaction between its different components, which hinders a more comprehensive understanding of PICS. Another important challenge is the lack of standardization in assessment tools, making comparison between studies difficult and limiting the applicability of scientific findings. To overcome these limitations, it is essential to strengthen research through international partnerships, promoting multicenter and longitudinal studies that assess not only the impact of PICS on patients, but also on their families and caregivers.

## Conclusion

This bibliometric study presents a comprehensive summary and analysis of emerging trends and themes in PICS, with an emphasis on patient follow-up after discharge from the ICU. There remains a concern for the early identification of risk factors associated with the development of PICS and effective preventive measures. Follow-up after discharge from the ICU is still fragmented, with several studies addressing only one component of PICS, rather than providing a holistic assessment of the patient, namely the integration of the domain of spirituality. It is essential to investigate and implement effective interventions in the treatment of the various dysfunctions associated with PICS, promoting quality care centered on the patient and their family, which facilitates the social and functional reintegration of both. 

We have not found an ideal method of follow-up after discharge from the ICU, nor comparative studies between the different methods: in person in a consultation setting, by telephone, specific clinics, or at home. Currently, there are several tools for screening for PICS, with some recent consensus proposing specific instruments for each component of the syndrome. However, there is still no single, validated instrument that assesses all dimensions in an integrated manner, limiting a systematic and comprehensive approach in clinical practice.

In the field of nursing, structured post-ICU follow-up programs are needed, focusing on functional assessment, emotional support, and education for self-management. Telephone follow-up and home visits can optimize continuity of care. Future research should evaluate the effectiveness of these interventions and their impact on patients’ quality of life and hospital readmissions.

Advances in research and the implementation of evidence-based strategies are essential to improving post-ICU care. To this end, well-designed future studies are needed to promote international cooperation and contribute to the development of more integrated and sustained clinical practices. 

## Data Availability

All data generated or analysed during this study are included in this published article.
