# Supplementary material for: Post-intensive care unit syndrome: trends and research foci in a bibliometric analysis
Source: Rev Lat Am Enfermagem. 2026 Oct 9;34:e4927. doi: 10.1590/1518-8345.8124.4927 (PMC13652530; doi:10.1590/1518-8345.8124.4927)
Supplement: Translation

# Translation: Síndrome pós-internamento em cuidados intensivos: tendências e focos de investigação numa análise bibliométrica

**DOI:** 10.1590/1518-8345.8124.4928

**Authors:** Natália Soares da Silva, Paulo Jorge Pereira Alves, Elsa Maria Oliveira Pinheiro de Melo, Rafael Alves Bernardes

**Affiliations:** 1 Universidade Católica Portuguesa, Faculdade de Ciências da Saúde e Enfermagem, Porto, Portugal.; 2 Universidade de Aveiro, Escola Superior de Saúde, Aveiro, Portugal.; 3 Universidade Católica Portuguesa, Centro de Investigação Interdisciplinar em Saúde, Lisboa, Portugal.; 4 Universidade de Aveiro, Unidade de Investigação Instituto de Biomedicina, Aveiro, Portugal.; 5 Universidade Católica Portuguesa, Faculdade de Ciências da Saúde e Enfermagem, Lisboa, Portugal.

**Keywords:** Bibliometria, Cuidados Críticos, Guia de Prática Clínica, Qualidade de Vida, Seguimentos, Enfermagem de Cuidados Críticos

## Abstract

**Objetivo:**

identificar os principais focos e tendências na pesquisa sobre Síndrome Pós-Internamento em Cuidados Intensivos, com ênfase no seguimento dos pacientes após a alta da Unidade de Cuidados Intensivos.

**Método:**

pesquisa realizada na Web of Science no período de 2012 a novembro de 2024. Utilizou-se o pacote Bibliometrix e a plataforma BiblioShiny no RStudio para explorar as palavras-chave, tendências e coocorrências, revistas, autores, cocitações e redes de colaboração. 120 artigos sobre Síndrome Pós-Internamento em Cuidados Intensivos em adultos foram incluídos.

**Resultados:**

as principais palavras-chave incluem *critical illness*, *quality-of-life* e *posttraumatic stress disorder*. Os autores mais produtivos são Needham D.M., Sevin C.M., e os periódicos mais relevantes são *Critical Care* e *BMJ Open*. Os Estados Unidos lideram as publicações, seguidos pelos Países Baixos e Japão.

**Conclusão:**

a maioria dos estudos aborda um único componente da síndrome, a comparação entre estudos é dificultada pela diversidade de instrumentos de avaliação. É necessária maior colaboração internacional, estudos a longo prazo, Ensaios Clínicos Randomizados e Controlados.

## Destaques

**(1)** A investigação em SPICI cresceu, com foco em reabilitação e qualidade de vida.

**(2)** Estudos abordam isoladamente domínios da SPICI, limitando abordagens integradas.

**(3)** Faltam consensos e ferramentas padronizadas para rastreio e intervenção eficaz.

## Introdução

Os avanços na Medicina Intensiva, nomeadamente a implementação de diretrizes terapêuticas baseadas em evidências e a qualificação contínua dos profissionais de saúde, têm contribuído significativamente para a redução da mortalidade a curto prazo em pacientes críticos^1^ ^-^ ^2^. No entanto, embora a sobrevivência hospitalar tenha melhorado substancialmente, a qualidade de vida e os resultados funcionais a longo prazo dos pacientes continuam a ser um desafio importante^2^ ^-^ ^4^.

Nos últimos anos, os profissionais de saúde têm mostrado uma preocupação crescente na compreensão das sequelas dos pacientes após a alta da Unidade de Cuidados Intensivos (UCI). A Síndrome Pós Internamento em Cuidados Intensivos (SPICI) foi descrita pela primeira vez pela Sociedade Americana de Medicina de Cuidados Críticos (SCCM), na Conferência Global de Cuidados Críticos em 2010, definindo-a como um conjunto de déficits físicos, cognitivos e psicológicos que podem afetar tanto os sobreviventes da UCI, quanto os seus familiares (*Post-Intensive Care Syndrome-Family* - PICS-F)^5^ ^-^ ^6^.

Dada as consequências da síndrome, torna-se essencial aprofundar o seu estudo, compreender os fatores de risco envolvidos e desenvolver estratégias eficazes de reabilitação para minimizar os seus efeitos adversos^6^. A investigação contínua sobre intervenções multidisciplinares, incluindo programas de reabilitação precoce, suporte psicológico e monitorização pós-alta pode desempenhar um papel essencial na melhoria dos resultados para os doentes e suas famílias^4^.

A Síndrome Pós Internamento em Cuidados Intensivos compreende seis atributos definidores: compromissos multidimensionais, novos ou agravados; disfunção física; transtorno psicológico; compromisso a nível cognitivo; reconstrução social falhada e sintomas prejudicados que persistem após a alta da UCI^7^ ^).^

Trata-se de uma síndrome complexa, na qual interagem múltiplas dimensões, afetando significativamente a qualidade de vida e a reintegração social dos sobreviventes^8^ ^-^ ^10^.

A SPICI apresenta uma prevalência elevada, sendo, por isso, necessário acompanhamento a longo prazo em regime ambulatorial após a alta. A sua prevalência varia amplamente conforme a população estudada, os critérios diagnósticos utilizados e as condições de seguimento pós-alta. De todos os pacientes que sobrevivem a um internamento em UCI, aproximadamente 50% terão pelo menos um dos problemas associados à SPICI^11^. Em alguns casos, 64,9% deles apresentam sintomas logo na primeira consulta após a alta UCI^9^.

Para a avaliação da SPICI, as recomendações baseadas em consenso enfatizam a avaliação da cognição, saúde mental e função física utilizando várias ferramentas^5^ ^,^ ^8^ ^,^ ^12^.

Os fatores de risco para SPICI são classificados em antecedentes do utente, gravidade da doença, cuidados médicos e estresse do ambiente^13^. A idade avançada, sexo feminino, problemas de saúde mental anteriores, gravidade da doença, experiência negativa em internamento de cuidados intensivos e *delirium* representam fatores de risco significativos para SPICI^14^. É de se fazer notar que um dos fatores associados a um risco aumentado de desenvolver comprometimento cognitivo pós internamento em UCI incluiu a ocorrência de *delirium* ^4^ ^,^ ^9^ ^,^ ^15^.

Os antecedentes, em pacientes com internamento em UCI, são os principais contribuintes para a ocorrência de SPICI, seja em decorrência dos tratamentos específicos ou da própria gravidade da doença crítica^7^.

A experiência vivida por familiares e/ou cuidadores informais de pessoas em estado crítico pode desencadear sintomas psicológicos significativos. Nesse contexto, é essencial reconhecer que a saúde desses cuidadores é um componente crucial do processo terapêutico. A Síndrome Pós Internamento em Cuidados Intensivos Familiar (SPICI-F) foi observada em 19,1% dos familiares e/ou cuidadores, sendo que a alteração mais frequente é o aumento da sobrecarga familiar e a ansiedade^9^. Além disso, os próprios familiares experimentam períodos de estresse e ansiedade contínuos após a alta do familiar^16^.

A prevenção da SPICI está intimamente relacionada à identificação precoce de potenciais fatores de risco modificáveis. A aplicação de um plano de cuidados agrupado como o *bundle* ABCDEF (analgesia e seleção da sedação, provas de ventilação espontânea, coordenação entre as provas de ventilação espontânea e despertar, prevenção do *delirium*, exercício e mobilização/reabilitação precoce e, finalmente, comunicação e envolvimento dos familiares), pode melhorar o cuidado centrado no paciente, bem como a experiência dos doentes e suas famílias^9^. 

Uma experiência negativa do paciente em internamento na UCI pode ser modificada através da implementação de estratégias para reduzir o *delirium*, aumentar a mobilização precoce, otimizar o controle da dor e reduzir ou modificar o uso de restrições físicas^17^.

Não há consenso na literatura sobre o modelo ideal de seguimento da pessoa pós-doença crítica. Contudo, deve-se observar três pilares: a identificação de risco de SPICI e sua prevenção; a identificação da SPICI nas suas múltiplas componentes e a abordagem multiprofissional^18^. 

Dada a escassez de literatura específica e a ausência de diretrizes consolidadas sobre a SPICI, surge a necessidade de mapear a profundidade e a natureza das investigações sobre os desfechos de longo prazo, a eficácia das intervenções e a padronização metodológica. Assim, é objetivo deste estudo identificar os principais focos e tendências na pesquisa sobre Síndrome Pós-Internamento em Cuidados Intensivos, com ênfase no seguimento dos pacientes após a alta da Unidade de Cuidados Intensivos. 

## Método

### Desenho do estudo

Este estudo aplicou a análise bibliométrica como desenho de pesquisa, a qual foi reconhecida como uma metodologia eficaz para estudar quantitativamente a produção científica^19^ ^-^ ^20^. Essa abordagem permite identificar padrões, tendências e a evolução do conhecimento científico ao longo do tempo, analisando grandes conjuntos de dados de forma sistemática. A análise foi conduzida com base nas principais leis da bibliometria: a Lei de Bradford, aplicada para definir o núcleo de revistas mais produtivas e categorizar as demais em zonas de dispersão; a Lei de Lotka, utilizada para distribuir os autores segundo a sua produtividade, identificando os mais ativos no tema; e a Lei de Zipf, que orientou a depuração e agrupamento de termos, criando uma lista representativa dos conceitos mais frequentes nos artigos analisados^19^.

Para a análise, foram utilizadas as ferramentas Bibliometrix e BiblioShiny no *software* RStudio, desenvolvidas^20^ para estatísticas descritivas, e o *software* VOSviewer que permite a construção e visualização de mapas bibliométricos^21^ baseados em redes de coocorrência de palavras-chave, colaboração entre autores e citações.

Neste contexto, foram formuladas as seguintes questões de pesquisa: Quais são as palavras-chave mais relevantes usadas em estudos de pesquisa relacionados à SPICI? Qual é a tendência nos padrões da investigação, interesses emergentes e mudanças de práticas nos diferentes contextos de investigação ao longo dos anos? Quais são as principais fontes de publicação de artigos de pesquisa?

### *Screening* e análise de dados

Neste estudo de análise bibliométrica, dois revisores examinaram independentemente todas as publicações, procedendo à leitura de título e resumo. As discrepâncias foram resolvidas por consenso, e, nos casos em que não houve acordo, um terceiro revisor atuou como decisor. O processo de recolha das publicações está indicado na Figura 1. A pesquisa foi realizada na Web of Science à semelhança de outros estudos^22^ ^-^ ^23^, a 20 de novembro de 2024; assim, os dados referentes ao ano de 2024 são parciais. Esta base de dados fornece aos pesquisadores uma gama diversificada de publicações de alta qualidade^24^, com registros bibliográficos padronizados e consistência temporal, assegurando maior rigor, transparência e reprodutibilidade na análise bibliométrica. Reconhece-se, contudo, que a opção por uma única base pode limitar a representatividade dos resultados.

**Figura 1:** Fluxograma da revisão bibliométrica. Aveiro, Portugal, 2024

Os critérios de inclusão foram: (i) estudos publicados a partir de 2012, uma vez que foi o momento em que surgiu a primeira definição formal dessa síndrome^4^; (ii) artigos publicados em português, inglês e espanhol; (iii) estudos com foco na SPICI e/ou SPICI-F; (iv) investigação centrada no seguimento dos pacientes após a alta da UCI; (iv) estudos com população adulta (>18 anos). Os critérios de exclusão foram: literatura cinzenta, capítulos de livro, *proceedings*/*abstracts* de conferências.

Após a aplicação dos critérios de seleção, os documentos foram salvos no formato BibTeX. O arquivo final foi exportado para Excel para revisão manual. 

Reconhece-se que a estratégia de busca adotada é restritiva, podendo excluir alguns trabalhos relevantes, em futuros estudos pode ser melhorada ampliando sinônimos ou flexibilizando blocos.

### Análise estatística

Realizou-se uma análise de coocorrência no VOSviewer com as 50 principais *Author Keywords* dos 120 artigos, selecionadas segundo as zonas da Lei de Bradford e previamente normalizadas por meio da remoção de duplicados e unificação de sinônimos, com base numa revisão manual sistemática dos termos. A análise de rede fornece vários *clusters* na rede, representados por cores distintas, com uma frequência ≥ 5 no agrupamento de palavras-chave. O tamanho do nó circular no mapa é proporcional à frequência da palavra-chave, e a distância e a espessura das linhas entre as palavras indicam a força do seu relacionamento. Enquanto a distância geral entre as palavras-chave reflete a sua correlação, geralmente uma distância menor entre duas palavras-chave implica um relacionamento mais forte entre elas. A cor do nó mostra o *cluster* ao qual a palavra-chave está associada. Cada *cluster* pertence a um tema de pesquisa representado pelas palavras-chave e seus links.

Para a análise dos dados, utilizou-se o pacote Bibliometrix, desenvolvido na linguagem de programação R, dentro do ambiente RStudio. Esse pacote permitiu a extração e organização dos principais tópicos de investigação, autores mais produtivos, revistas de maior impacto, países com maior contribuição científica e colaborações internacionais na área de estudo. 

Além disso, foram identificados os artigos mais relevantes com base no número de citações, permitindo uma visão abrangente da influência acadêmica na área de SPICI.

A análise bibliométrica obedeceu a quatro elementos:

Análise de Coocorrência de Palavras-Chave: identificação de temas predominantes e tendências emergentes com base na frequência e no agrupamento de palavras-chave.

Análise de Citações: avaliação dos artigos, autores e revistas mais citados no conjunto de dados.

Análise da Rede de Coautoria: examinar as colaborações entre autores, instituições e países.

Análise de Cocitação: mapear as estruturas intelectuais através da análise de trabalhos frequentemente cocitados.

## Resultados

Os estudos relacionados com SPICI começaram a surgir formalmente em 2012 e, desde então, a produção científica tem mostrado um crescimento contínuo ao longo dos anos.

### Palavras-chave

A análise de palavras-chave e as suas tendências ao longo tempo permitem identificar padrões da investigação, interesses emergentes e mudanças de práticas nos diferentes contextos de investigação.

A partir de 2015, uma massa crítica de artigos existentes permitiu a identificação de palavras-chave com frequência mínima. A proeminência de palavras-chave específicas tornou-se evidente em 2021, com termos como “*posttraumatic-stress-disorder*”, “*quality-of-life*” e “*critical illness*”. Recentemente, os termos mais utilizados são “*impact”, “unit”* e *“rehabilitation*”, refletindo uma abordagem centrada nos desfechos pós-alta com foco na recuperação dos sobreviventes de doença crítica.

Na Figura 2, são apresentados os termos mais pesquisados, bem como as suas coocorrências, com uma frequência ≥ 5 coocorrências, fornecendo uma maior compreensão sobre o cenário de pesquisa.

O mapa de rede ilustra a estrutura altamente interconectada e multidisciplinar da pesquisa focada na “*post intensive care syndrome*”, que emerge como elemento central da rede, articulando-se com os principais temas da literatura. Entre estes, destaca-se “*critical illness*”, o foco mais robusto do campo, com 96 ocorrências e uma força de ligação total de 813. 

Fazem parte desta rede outros clusters temáticos (saúde mental, *delirium*/gestão clínica, reabilitação e função cognitiva), mostrando a complexidade do campo de estudo. O *cluster* vermelho concentra-se nas repercussões emocionais e nos determinantes psicossociais associados à PICS. Os estudos aqui reunidos enfatizam a elevada carga de morbilidade psicológica. A relevância do contexto social é evidenciada pela presença do termo “*caregivers*”, que surge cinco vezes, com uma força de ligação total de 41. Este *cluster* tem uma forte sinergia com o *cluster* roxo, sublinhando a importância de integrar o suporte familiar e a dinâmica dos cuidadores nas abordagens de acompanhamento “*follow-up*”.

**Figura 2:** Análise de coocorrência de termos de pesquisa. Aveiro, Portugal, 2024

O *cluster* verde reúne temas relacionados à fase aguda da doença crítica. O “*delirium*”, destaca-se como o tópico clínico mais proeminente, com 21 ocorrências e 175 de força de ligação total, refletindo o seu papel central como marcador de gravidade, complicação frequente e preditor de resultados físicos, cognitivos e psicológicos no período pós-alta.

O *cluster* azul é focado nas dimensões físicas da recuperação. Os conceitos de “*rehabilitation*” e “*recovery*” estruturam este grupo, mostrando a crescente atenção dada às intervenções de reabilitação física e ao restabelecimento funcional após a permanência na UCI.

Finalmente, o *cluster* amarelo abrange os efeitos cognitivos persistentes após a doença crítica, incluindo défices de memória, atenção e funções executivas. A “*quality of life*” surge como nó central, ligando as sequelas cognitivas e funcionais aos resultados gerais dos sobreviventes.

Os grupos de termos indicam uma transição da investigação de diagnóstico para a gestão integrada e personalizada, com foco crescente em mecanismos biológicos e na experiência do doente.

### Revistas

O número de artigos publicados em revistas especializadas é um indicador da relevância do tema dentro da comunidade científica. A Tabela 1 apresenta as revistas que mais artigos publicaram na área da SPICI, de acordo com o h-index e com o número total de citações.

**Tabela 1:** Análise de publicações: h_index e citações. Aveiro, Portugal, 2024

Revistas	h_index*	g_index^†^	*TC* ^*‡*^	*NP* ^*§*^	*PY-start* ^*||*^
*Critical Care*	7	10	543	10	2016
*BMJ Open*	6	8	201	8	2017
*Critical Care Medicin*e	5	8	800	8	2014
*Annals Of The American Thoracic Society*	3	4	160	4	2016
*Critical Care Explorations*	3	4	36	4	2022
*Current Opinion In Critical Care*	3	5	79	5	2012
*Intensive And Critical Care Nursing*	3	4	32	4	2020
*Journal Of Intensive Care*	3	3	52	3	2023
*Acute Medicine & Surgery*	2	2	8	2	2024
*Australian Critical Care*	2	3	24	3	2022
*Critical Care Nurs*e	2	2	8	2	2022
*Journal Of Advanced Nursin*g	2	2	8	2	2021
*Journal Of Clinical Medicine*	2	3	15	3	2021
*Journal Of Critical Care*	2	3	184	3	2015
Medicina Intensiva	2	2	17	2	2019

*h-index= Métrica que fornece uma visão equilibrada da produtividade e do impacto do autor; ^†^g-index = Índice que amplifica as citações dos artigos mais citados para destacar as distinções entre os investigadores; ^‡^TC = Total de citações; ^§^NP = Número de publicações; ^||^PY-start = Primeira publicação

A revista com mais publicações e maior relevância é a revista “*Critical Care”* que publicou entre 2016 e 2024, um total de 10 trabalhos, com 543 citações, e que apresenta um h- index de 7 e um g-index de 10.

Nessa lista, surgem oito revistas com a primeira publicação sobre a temática em análise a partir do ano 2020, evidenciando uma crescente consolidação do tema como campo de investigação relevante.

Além disso, é importante referir que dessa lista fazem parte três revistas de enfermagem, “*Intensive And Critical Care Nursing”, “Critical Care Nurse”* e *“Journal of Advanced Nursing*”, o que fornece informações sobre o impacto e a consistência das publicações dessas revistas no âmbito de enfermagem e cuidados intensivos.

### Análise de autores e citações

A Tabela 2 sumariza os autores no que respeita aos contributos dados no domínio da investigação da SPICI.

**Tabela 2:** Análise de citações - avaliação autores. Aveiro, Portugal, 2024

Autores		h_index*	g_index^†^	*TC* ^*‡*^	NP^§^	PY_start^||^
Needham D.M.		8	12	808	12	2014
Sevin C.M.		7	9	501	9	2018
Hopkins R.O.		6	7	668	7	2012
Jackson J.C.		6	7	765	7	2018
Akhlaghi N.		4	4	150	4	2019
Bose S.		4	4	150	4	2019
Brown S.M.		4	4	150	4	2019
Dinglas V.D.		4	5	150	5	2019
Ely E.W.		4	4	434	4	2018

* *h-index=* Métrica que fornece uma visão equilibrada da produtividade e do impacto do autor*;* ^*†*^ g-index = Índice que amplifica as citações dos artigos mais citados para destacar as distinções entre os investigadores*;* ^*‡*^ TC *=* Total de citações*;* ^*§*^ NP *=* Número de publicações*;* ^*||*^ PY_start *=* Primeira publicação

Avaliado em termos de número de artigos que publicaram, h-índex, g-índex e número total de citações obtidas, destaca-se Needham, com 12 publicações e com o maior número de citações: 808, que juntamente com Hopkins são dos autores que publicam há mais tempo sobre a temática. 

Com publicações mais recentes, desde 2018, destacam-se Sevin com nove publicações e 501 citações, ou seja, o impacto das suas publicações é bem representado pelo número de citações dos seus artigos mais citados, e Jackson, que apresenta 7 artigos com um total de 765 citações e h-índex de 6; este autor tem uma base sólida de trabalhos bem citados, com um impacto significativo.

A Figura 3 apresenta a lista dos artigos mais citados, identificando os autores, o ano de publicação, o título do artigo e o número de citações. 

Dos artigos mais citados, destacam-se “*Co-occurrence of Post-Intensive Care Syndrome Problems Among406 Survivors of Critical Illness”* e *“Anxiety, Depression and Post Traumatic Stress Disorder after critical illness: a UK wide prospective cohort study”*, ambos de 2018.

**Figura 3:** Análise de citações. Aveiro, Portugal, 2024

Autor, ano	Título	Citações
Marra, et al., 2018	*Co-occurrence of Post-Intensive Care Syndrome Problems Among 406 Survivors of Critical Illness*	287
Elliott, et al., 2014	*Exploring the Scope of Post-Intensive Care Syndrome Therapy and Care: Engagement of Non-Critical Care Providers and Survivors in a Second Stakeholders Meeting*	281
Hatch, et al., 2018	*Anxiety, Depression and Post Traumatic Stress Disorder after critical illness: a UK wide prospective cohort study*	281
Mikkelsen, et al., 2020	*Society of Critical Care Medicine’s International Consensus Conference on Prediction and Identification of Long-Term Impairments After Critical Illness*	202
Fuke, et al., 2018	*Early rehabilitation to prevent postintensive care syndrome in patients with critical illness: a systematic review and meta-analysis*	130
Sevin, et al., 2018	*Comprehensive Care of ICU Survivors: Development and Implementation of an ICU Recovery Center*	126
Brown, et al., 2019	*Approaches to Addressing Post-Intensive Care Syndrome among Intensive Care Unit Survivor*	126
Gayat, et al., 2018	*Determinants of long-term outcome in ICU survivors: results from the FROG-ICU study*	124
Schofield-Robinson, et al., 2018	*Follow-up services for improving long-term outcomes in intensive care unit (ICU) survivors (Review)*	121
Jensen, et al., 2015	*Impact of follow-up consultations for ICU survivors on post-ICU syndrome: a systematic review and meta-analysis*	110
Major, et al.,2016	*Surviving critical illness: what is next? An expert consensus statement on physical rehabilitation after hospital discharge*	80

O artigo^25^ “*Exploring the Scope of Post-Intensive Care Syndrome Therapy and Care: Engagement of Non-Critical Care Providers and Survivors in a Second Stakeholders Meeting”*, com 281 citações, contribuiu para aumentar a conscientização e a educação sobre SPICI.

Com mais de 100 citações e publicado em 2019, encontra-se o artigo^26^ “*Approaches to Addressing Post-Intensive Care Syndrome among Intensive Care Unit Survivor”.*

### Distribuição da produção científica

A distribuição da produção científica sobre a SPICI, considerando tanto colaborações internacionais quanto publicações de autoria nacional, revelaram que os Estados Unidos lideram significativamente tanto em número absoluto de publicações quanto em colaborações internacionais, o que evidencia a sua centralidade na pesquisa sobre esta temática.

Os Países Baixos e o Japão aparecem logo em seguida, mostrando um forte envolvimento na produção científica e destacando-se pela ativa rede de colaboração global. Já a China, apesar de apresentar um volume considerável de publicações, não estabeleceu colaborações internacionais significativas dentro da amostra analisada. 

Os resultados sugerem que a produção científica sobre SPICI está concentrada em determinados polos académicos, sendo impulsionada por países que mostram maior investimento na investigação sobre cuidados pós-alta da UCI, incluindo reabilitação.

## Discussão

A análise bibliométrica, apesar de ter sido realizada apenas na Web of Science, permitiu responder às questões de investigação formuladas e fornecer um panorama abrangente sobre a evolução da pesquisa relacionada à SPICI, assim como verificar uma tendência crescente em artigos de pesquisa originais e revisões sobre o SPICI. Contudo esta limitação, bem como o número reduzido de artigos, pode introduzir viés de seleção reduzindo a generalização dos resultados.

A análise dos termos mais utilizados na investigação permite fornecer sugestões valiosas sobre o cenário da pesquisa^20^ ^,^ ^27^, é possível observar o aparecimento de termos emergentes como “*impact*” e “*rehabiliation*”. Esses termos refletem a transição da pesquisa inicial, que enfatizava a caracterização da síndrome, para uma abordagem mais aplicada, voltada para a reabilitação e qualidade de vida, que é afetada significativamente após internamento na UCI^8^ ^,^ ^19^. Os elementos dos *clusters* confirmam a complexidade multidimensional da Síndrome Pós-Cuidados Intensivos, ampliam o entendimento com novas dimensões e tecnologias, e questionam o modelo de seguimento pós internamento em Cuidados Intensivos e a heterogeneidade metodológica.

A evolução da investigação ao longo dos anos mostrou um aumento do interesse por temas como transtorno de estresse pós-traumático, qualidade de vida e impacto da doença crítica, destacando a necessidade de abordagens holísticas e intervenções multidisciplinares que facilitem a adaptação do doente à nova situação de saúde. O conhecimento descritivo amadureceu, mas a investigação interventiva, preditiva e translacional permanece pouco desenvolvida.

A bibliometria revelou que a investigação sobre SPICI está concentrada em revistas científicas de grande relevância na área de cuidados críticos e medicina intensiva, com destaque para as revistas: *Critical Care* ^22^ *, BMJ Open* e *Critical Care Medicine* ^23^, que apresentaram os maiores índices de produtividade e citações. A presença de três periódicos específicos de enfermagem na lista dos mais relevantes demonstra a importância crescente que os enfermeiros atribuem à deteção, gestão e reabilitação dos sobreviventes da UCI.

Os enfermeiros desempenham um papel fundamental na minimização da SPICI, através de intervenções focadas na mobilização precoce, gestão do *delirium*, apoio à família e planeamento da alta^28^. Também no acompanhamento dos sobreviventes e família, as intervenções de enfermagem visam ativar estratégias eficazes de enfrentamento, promoção do autocuidado, recuperação da autonomia do doente e prevenção da fadiga associada ao papel do cuidador^29^. O acompanhamento dos pacientes após o internamento em UCI representa uma área emergente no âmbito dos cuidados de saúde, visam identificar e abordar as consequências a longo prazo da permanência em UCI de forma mais eficaz, do que os cuidados-padrão^30^. Contudo, os dados disponíveis são limitados quanto às intervenções eficazes a serem implementadas e evidenciam a falta de padronização de um modelo de seguimento dos doentes após doença crítica^30^, com lacunas na implementação de abordagens integradas e longitudinais. 

Identificou-se uma alta prevalência de comprometimento cognitivo, ansiedade, depressão, debilidade física, mudanças no estilo de vida e problemas relacionados com a medicação que justificam intervenção nos doentes pós internamento em cuidados intensivos^31^. A SPICI está associada a uma diminuição da qualidade de vida dos doentes após a alta da UCI^9^. O conceito do “eu alterado”, é a transformação do *self* refletindo as novas limitações físicas vivenciadas, assim como a maneira pela qual a mobilização das características intrínsecas do indivíduo facilita a adaptação à sua nova identidade^32^.

O acompanhamento dos doentes pode ajudar a criar a sua narrativa de doença e a recuperar um sentido de coerência, contudo são necessários ensaios clínicos aleatorizados que padronizem as intervenções e as medidas de resultados^33^.

Os autores^5^ recomendam avaliações sistematizadas e sugerem algumas ferramentas de rastreio para avaliar cada uma das componentes de SPICI, destacando a importância de priorizar doentes de alto risco, e encaminhar os mesmos para serviços e/ou avaliações mais detalhadas. É necessária uma avaliação sistemática e frequente da SPICI em todo o contínuo de cuidados^25^.

É importante identificar fatores de risco para o desenvolvimento de SIPCI, e a equipe multidisciplinar deve estar atenta a fatores de risco modificáveis. O aumento da compreensão dos fatores de risco melhorou a capacidade de identificar doentes de alto risco para rastreio e intervenção^17^. Os autores^10^ avaliaram a incidência de SPICI em sobreviventes de COVID-19 e identificaram o *delirium* e a traqueostomia como fatores associados. A equipe de enfermagem desempenha um papel preponderante, quer na identificação precoce dos fatores de risco, quer na implementação de *bundles,* diminuindo o risco de desenvolvimento de SPICI.

É importante a realização de estudos que examinem a implementação de recomendações, incluindo a sua viabilidade, aceitabilidade, sustentabilidade e fidelidade, para além de avaliarem o impacto das recomendações nos resultados a longo prazo para os sobreviventes^5^. A investigação deve ter como foco os pacientes e a transições de cuidados^26^, sendo que a transição eficaz dos cuidados deve assegurar uma continuidade entre o tratamento na UCI, na enfermaria e o apoio contínuo na comunidade e domicílio.

A análise bibliométrica revelou, ainda, desigualdades na distribuição da produção científica sobre SPICI e áreas relacionadas entre países e continentes; destaca-se, adicionalmente, a restrição linguística adotada (português, inglês e espanhol) como aspeto metodológico relevante da investigação. À semelhança de outros estudos^22^ ^-^ ^23^, os Estados Unidos lideram significativamente tanto em número de publicações quanto em colaborações internacionais, o que pode estar relacionado ao investimento robusto em cuidados pós-UCI e investigação longitudinal. Os Países Baixos e o Japão também se destacam como polos ativos de investigação, refletindo o desenvolvimento de estratégias avançadas de reabilitação e reestruturação dos cuidados intensivos.

Por outro lado, a China, apesar de possuir um volume expressivo de publicações, mostrou baixa colaboração internacional, possivelmente devido a barreiras linguísticas ou um modelo de investigação mais voltado para a realidade clínica interna. Portugal, por sua vez, evidencia uma participação reduzida e ausência de colaborações internacionais na amostra analisada. Isso pode ser atribuído a dificuldades no financiamento e à falta de redes colaborativas com grupos internacionais de cuidados intensivos nomeadamente na Europa.

A SPICI continua a ser uma preocupação crescente, e há um consenso na literatura sobre a necessidade de aprimorar os mecanismos de identificação precoce e acompanhamento dos sobreviventes de doença crítica. Avaliações sistemáticas e frequentes da síndrome devem ser realizadas ao longo de todo o contínuo de cuidados, dando prioridade a doentes de alto risco para intervenção precoce^5^, e investigando a sua biologia molecular ou predisposições genéticas^22^. 

Estudos recentes apontam para a necessidade de estratégias de transição eficazes, garantindo uma continuidade nos cuidados entre a alta da UCI e o retorno à vida quotidiana^26^. O enfermeiro como coordenador deste acompanhamento é crucial devido à sua experiência na gestão de cuidados complexos e à sua capacidade de articulação entre o paciente , a família e a equipe multiprofissional^29^.

A crescente atenção dada à SPICI destaca a importância de ampliar as colaborações internacionais, acelerar a pesquisa em áreas relacionadas^22^ promovendo redes multicêntricas de investigação e garantindo que novas evidências científicas sejam aplicáveis a diversos cenários clínicos. O fortalecimento dessas redes pode contribuir para uma abordagem mais equitativa e eficaz do seguimento após internamento em UCI, beneficiando não apenas os doentes, mas também as suas famílias e os profissionais envolvidos na sua recuperação/reabilitação.

A investigação sobre SPICI apresenta limitações, focando-se apenas nos efeitos clínicos a curto prazo, com pouca análise a longo prazo e estudos multinacionais.Além disso, muitos estudos analisam apenas uma dimensão da síndrome, sem explorar a interação entre os seus diferentes componentes, o que dificulta uma compreensão mais abrangente da SPICI. Outro desafio importante é a falta de padronização nos instrumentos de avaliação, tornando a comparação entre estudos difícil e limitando a aplicabilidade dos achados científicos. Para superar essas limitações, é essencial fortalecer a investigação com parcerias internacionais, promovendo pesquisas multicêntricas e longitudinais que avaliem não apenas o impacto da SPICI no doente, mas também nos seus familiares e cuidadores.

## Conclusão 

Este estudo bibliométrico apresenta um resumo abrangente e uma análise das tendências e dos temas emergentes na SPICI com ênfase no seguimento dos pacientes após a alta da UCI. Mantêm-se a preocupação na identificação precoce de fatores de risco associados ao desenvolvimento de SPICI e nas medidas preventivas eficazes. O seguimento após alta da UCI apresenta-se ainda fragmentado, com diversos estudos abordando apenas uma das componentes de SPICI, não fazendo uma avaliação holística do doente, nomeadamente a integração do domínio da espiritualidade. Torna-se essencial investigar e implementar intervenções eficazes no tratamento das diversas disfunções associadas à SPICI, promovendo um cuidado de qualidade centrado no paciente e na sua família, que facilite a reintegração social e funcional de ambos. 

Não encontramos um método ideal de acompanhamento após a alta da UCI, nem estudos comparativos entre os diferentes métodos: presencial em contexto de consulta, telefónica, clínicas especificas, domicílio. Atualmente, existem diversas ferramentas para o rastreio da SPICI, com alguns consensos recentes a proporem instrumentos específicos para cada componente da síndrome. No entanto, permanece inexistente um instrumento único e validado que avalie de forma integrada todas as dimensões limitando a abordagem sistematizada e abrangente na prática clínica.

No domínio da enfermagem são necessários programas estruturados de acompanhamento pós-UCI, com foco na avaliação funcional, apoio emocional e educação para a autogestão. O seguimento telefónico e as visitas domiciliárias podem otimizar a continuidade dos cuidados. Futuras investigações deverão avaliar a eficácia dessas intervenções e o seu impacto na qualidade de vida e readmissões hospitalares dos doentes.

O avanço da investigação e a implementação de estratégias baseadas em evidência são fundamentais para melhorar os cuidados pós-UCI. Para tal, são necessários estudos futuros bem delineados, que promovam a cooperação internacional e contribuam para o desenvolvimento de práticas clínicas mais integradas e sustentadas. 

## Data Availability

Todos os dados gerados ou analisados durante este estudo estão incluídos neste artigo publicado.

# Translation: Síndrome Post-Cuidados Intensivos: tendencias y enfoques de investigación en un análisis bibliométrico.

**DOI:** 10.1590/1518-8345.8124.4926

**Authors:** Natália Soares da Silva, Paulo Jorge Pereira Alves, Elsa Maria Oliveira Pinheiro de Melo, Rafael Alves Bernardes

**Affiliations:** 1 Universidade Católica Portuguesa, Faculdade de Ciências da Saúde e Enfermagem, Porto, Portugal.; 2 Universidade de Aveiro, Escola Superior de Saúde, Aveiro, Portugal.; 3 Universidade Católica Portuguesa, Centro de Investigação Interdisciplinar em Saúde, Lisboa, Portugal.; 4 Universidade de Aveiro, Unidade de Investigação Instituto de Biomedicina, Aveiro, Portugal.; 5 Universidade Católica Portuguesa, Faculdade de Ciências da Saúde e Enfermagem, Lisboa, Portugal.

**Keywords:** Bibliometría, Cuidados Críticos, Guía de Práctica Clínica, Calidad de Vida, Estudios de Seguimiento, Enfermería de Cuidados Críticos

## Abstract

identificar los principales enfoques y tendencias en la investigación sobre el Síndrome Post-Cuidados Intensivos, con énfasis en el seguimiento de los pacientes tras el alta de la Unidad de Cuidados Intensivos.

**Método:**

investigación realizada en la Web of Science de 2012 hasta noviembre de 2024. Se utilizó el paquete Bibliometrix y la plataforma BiblioShiny en el estudio para explorar palabras clave, tendencias y coocurrencias, revistas, autores, cocitaciones y redes de colaboración. Se incluyeron 120 artículos sobre el Síndrome Post-Cuidados Intensivos en adultos.

**Resultados:**

las principales palabras clave incluyen *critical illness*, *quality-of-life* e *posttraumatic stress disorder*. Los autores más prolíficos son Needham DM, Sevin CM, y las revistas más relevantes son *Critical Care* and *BMJ Open*. Estados Unidos lidera las publicaciones, seguido de los Países Bajos y Japón.

**Conclusión:**

la mayoría de los estudios abordan un solo componente del síndrome, la diversidad de instrumentos de evaluación dificulta la comparación entre estudios. Se necesita mayor colaboración internacional, estudios a largo plazo y ensayos clínicos controlados aleatorizados.

## Destacados

**(1)** La investigación en SPCI creció, con enfoque en la rehabilitación y la calidad de vida.

**(2)** Los estudios abordan de forma aislada los dominios de la SPCI, limitando abordajes integrados.

**(3)** Faltan consensos y de herramientas estandarizadas para una detección e intervención efectivas.

## Introducción

Los avances en Medicina Intensiva, en particular la implementación de directrices terapéuticas basadas en la evidencia y la capacitación continua de los profesionales sanitarios, han contribuido significativamente a la reducción de la mortalidad a corto plazo en pacientes críticos^1^ ^-^ ^2^. Sin embargo, aunque la supervivencia hospitalaria ha mejorado sustancialmente, la calidad de vida y los resultados funcionales a largo plazo de los pacientes siguen siendo un desafío importante^2^ ^-^ ^4^.

En los últimos años, los profesionales sanitarios han mostrado una creciente preocupación por comprender las secuelas que sufren los pacientes tras el alta de la Unidad de Cuidados Intensivos (UCI). El Síndrome Post-Cuidados Intensivos (SPCI) fue descrito por primera vez por la Sociedad Americana de Medicina de Cuidados Críticos (SCCM) en la Conferencia Global de Cuidados Críticos de 2010, definiéndolo como un conjunto de déficits físicos, cognitivos y psicológicos que pueden afectar tanto a los supervivientes de la UCI como a sus familias (*Post-Intensive Care Syndrome-Family* - PICS-F)^5^ ^-^ ^6^.

Dadas las consecuencias del síndrome, es fundamental profundizar en su estudio, comprender los factores de riesgo implicados y desarrollar estrategias de rehabilitación eficaces para minimizar sus efectos adversos^6^. La investigación en curso sobre intervenciones multidisciplinarias, incluyendo programas de rehabilitación precoz, apoyo psicológico y seguimiento tras el alta, puede desempeñar un papel esencial en la mejora de los resultados para los pacientes y sus familias^4^.

El síndrome Post-Cuidados Intensivos comprende seis atributos definitorios: deficiencias multidimensionales nuevas o agravadas; disfunción física; trastorno psicológico; deterioro cognitivo; reconstrucción social fallida y síntomas deteriorados que persisten tras el alta de la UCI^7^ ^)^ .

Se trata de un síndrome complejo, en el que interactúan múltiples dimensiones, afectando significativamente la calidad de vida y la reintegración social de los sobrevivientes^8^ ^-^ ^10^.

La SPCI tiene una alta prevalencia, por lo que es necesario un seguimiento ambulatorio a largo plazo tras el alta. Su prevalencia varía considerablemente según la población estudiada, los criterios diagnósticos utilizados y las condiciones de seguimiento tras el alta. De todas las personas que sobreviven a un ingreso en la UCI, aproximadamente el 50% presentará al menos uno de los problemas asociados al SPCI^11^. En algunos casos, el 64,9% de los pacientes presentan síntomas ya en la primera consulta tras el alta de la UCI^9^.

Para la evaluación del SPCI, las recomendaciones basadas en consenso enfatizan la evaluación de la cognición, la salud mental y la función física utilizando varias herramientas^5^ ^,^ ^8^ ^,^ ^12^.

Los factores de riesgo para SPCI se clasifican en función de los antecedentes del paciente, la gravedad de la enfermedad, los cuidados médicos y el estrés ambiental^13^. La edad avanzada, el sexo femenino, los problemas de salud mental previos, la gravedad de la enfermedad, la experiencia negativa en la unidad de cuidados intensivos y el *delirium* representan factores de riesgo significativos para SPCI^14^. Cabe destacar que uno de los factores asociados con un mayor riesgo de desarrollar deterioro cognitivo después del ingreso a la UCI incluyó la aparición de *delirium* ^4^ ^,^ ^9^ ^,^ ^15^.

Los antecedentes, en pacientes con ingreso en la UCI, son los principales contribuyentes de la aparición del SPCI, ya sea como consecuencia de los tratamientos específicos o de la propia gravedad de la enfermedad crítica^7^.

La experiencia vivida por familiares y/o cuidadores informales de personas en estado crítico puede desencadenar síntomas psicológicos significativos. En este contexto, es fundamental reconocer que la salud de estos cuidadores es un componente crucial del proceso terapéutico. El Síndrome Post-Cuidados Intensivos Familiares (SPCI-F) se observó en el 19,1% de los familiares y/o cuidadores, siendo la alteración más frecuente el aumento de la sobrecarga familiar y la ansiedad^9^. Además, los propios familiares experimentan períodos de estrés y ansiedad continuos tras el alta del familiar^16^.

La prevención del SPCI está íntimamente relacionada con la identificación precoz de potenciales factores de riesgo modificables. La aplicación de un plan de cuidados agrupado en el *bundle* ABCDEF (analgesia y selección de la sedación, pruebas de ventilación espontánea, coordinación entre las pruebas de ventilación espontánea y el despertar, prevención del delirium, ejercicio y movilización/rehabilitación precoz y, finalmente, comunicación e implicación de los familiares), puede el cuidado centrado en el paciente, así como la experiencia de los pacientes y sus familias^9^.

Una experiencia negativa del paciente ingresado a la UCI puede ser modificada a través de la implementación de estrategias para reducir el *delirium*, aumentar la movilización precoz, optimizar el control del dolor y reducir o modificar el uso de restricciones físicas^17^.

No existe consenso en la literatura sobre el modelo ideal de seguimiento para pacientes tras una enfermedad crítica. Sin embargo, se deben considerar tres pilares: la identificación del riesgo de SPCI y su prevención; la identificación del SPCI en sus múltiples componentes y el abordaje multiprofesional^18^.

Dada la escasez de literatura específica y la ausencia de directrices consolidadas sobre el SPCI, surge la necesidad de mapear la profundidad y la naturaleza de las investigaciones sobre los resultados a largo plazo, la eficacia de las intervenciones y la estandarización metodológica. Por lo tanto, el objetivo de este estudio es identificar los principales enfoques y tendencias en la investigación sobre el Síndrome Post-Cuidados Intensivos, con énfasis en el seguimiento de los pacientes tras el alta de la Unidad de Cuidados Intensivos.

## Método

### Diseño del estudio

Este estudio aplicó el análisis bibliométrico como diseño de investigación, el cual ha sido reconocido como una metodología eficaz para estudiar cuantitativamente la producción científica^19^ ^-^ ^20^. Este abordaje permite identificar patrones, tendencias y la evolución del conocimiento científico a lo largo del tiempo, mediante el análisis sistemático de grandes conjuntos de datos. El análisis se realizó con base en las principales leyes de la bibliometría: la Ley de Bradford, aplicada para definir el núcleo de las revistas más productivas y categorizar las demás en zonas de dispersión; la Ley de Lotka , utilizada para distribuir a los autores según su productividad, identificando a los más activos en el tema; y la Ley de Zipf , que orientó la depuración y agrupamiento de términos, creando una lista representativa de los conceptos más frecuentes en los artículos analizados^19^.

Para el análisis, se utilizaron las herramientas Bibliometrix y BiblioShiny en el *software* RStudio, desarrollado^20^ ^)^ para estadística descriptiva, y el *software* VOSviewer que permite la construcción y visualización de mapas bibliométricos^21^ ^)^ basados en redes de coocurrencia de palabras clave, colaboración entre autores y citaciones.

En este contexto se formularon las siguientes preguntas de investigación: ¿Cuáles son las palabras clave más relevantes utilizadas en estudios de investigación relacionados con SPCI? ¿Cuáles son las tendencias en los patrones de investigación, los intereses emergentes y los cambios en las prácticas en diferentes contextos de investigación a lo largo de los años? ¿Cuáles son las principales fuentes de publicación de artículos de investigación?

### Cribado y análisis de datos

En este estudio de análisis bibliométrico, dos revisores examinaron independientemente todas las publicaciones, realizando la lectura del título y resumen. Las discrepancias se resolvieron por consenso y, en caso de desacuerdo, un tercer revisor tomó la decisión. El proceso de recopilación de publicaciones se muestra en la Figura 1. La búsqueda se realizó en la Web of Science, al igual que en otros estudios^22^ ^-^ ^23^, el 20 de noviembre de 2024; por lo tanto, los datos correspondientes para el año 2024 son parciales. Esta base de datos proporciona a los investigadores una amplia gama de publicaciones de alta calidad^24^, con registros bibliográficos estandarizados y consistencia temporal, asegurando mayor rigor, transparencia y reproducibilidad en el análisis bibliométrico. Sin embargo, se reconoce que optar por una única base de datos puede limitar la representatividad de los resultados.

**Figura 1:** Flujograma de la revisión bibliométrica. Aveiro, Portugal, 2024

Los criterios de inclusión fueron: (i) estudios publicados a partir de 2012, ya que fue cuando surgió la primera definición formal de este síndrome^4^; (ii) artículos publicados en portugués, inglés y español; (iii) estudios con foco en el SPCI y/o SPCI-F; (iv) investigación centrada en el seguimiento de pacientes tras el alta de la UCI; (iv) estudios con población adulta (>18 años).

Los criterios de exclusión fueron: literatura gris, capítulos de libros, *proceedings*/*abstracts* de conferencias.

Tras la aplicación de los criterios de selección, los documentos se guardaron en formato BibTeX. El archivo final se exportó a Excel para su revisión manual.

Se reconoce que la estrategia de búsqueda adoptada es restrictiva, pudiendo excluir algunos estudios relevantes; en futuros estudios, podrá mejorarse mediante la ampliación de sinónimos o flexibilizando los bloques de búsqueda.

### Análisis estadístico

Se realizó un análisis de coocurrencia en VOSviewer con los 50 principales *Author Keywords* de los 120 artículos, seleccionados según las zonas de la Ley de Bradford y previamente normalizadas mediante la eliminación de duplicados y la unificación de sinónimos, con base en una revisión manual sistemática de los términos. El análisis de red proporciona varios clústeres en la red, representados por colores distintos, con una frecuencia ≥ 5 en la agrupación de palabras clave. El tamaño del nodo circular en el mapa es proporcional a la frecuencia de la palabra clave, y la distancia y el grosor de las líneas entre las palabras indican la fuerza de su relación. Mientras que la distancia general entre palabras clave refleja su correlación; generalmente, una distancia menor entre dos palabras clave implica una relación más fuerte entre ellas. El color del nodo muestra el clúster al que está asociada la palabra clave. Cada clúster pertenece a un tema de investigación representado por las palabras clave y sus enlaces.

Para el análisis de los datos, se utilizó el paquete Bibliometrix, desarrollado en el lenguaje de programación R, dentro del entorno RStudio. Este paquete permitió la extracción y organización de los principales temas de investigación, los autores más productivos, las revistas con mayor impacto, los países con mayor contribución científica y las colaboraciones internacionales en el área de estudio.

Además, se identificaron los artículos más relevantes con base en el número de citaciones, permitiendo una visión integral de la influencia académica en el campo de SPCI.

El análisis bibliométrico siguió cuatro elementos:

Análisis de coocurrencia de palabras clave: identificación de temas predominantes y tendencias emergentes basadas en la frecuencia y agrupamiento de palabras clave.

Análisis de citas: evaluación de los artículos, autores y revistas más citados en el conjunto de datos.

Análisis de la red de coautoría: examinar las colaboraciones entre autores, instituciones y países.

Análisis de cocitación: mapear las estructuras intelectuales a través del análisis de trabajos frecuentemente cocitados.

## Resultados

Los estudios relacionados con SPCI comenzaron a surgir formalmente en 2012 y, desde entonces, la producción científica ha mostrado un crecimiento continuo a lo largo de los años.

### Palabras clave

El análisis de palabras clave y sus tendencias a lo largo del tiempo nos permite identificar patrones de investigación, intereses emergentes y cambios en las prácticas en diferentes contextos de investigación.

A partir de 2015, una masa crítica de artículos existentes permitió la identificación de palabras clave con una frecuencia mínima. La prominencia de palabras clave específicas se hizo evidente en 2021, con términos como “*posttraumatic-stress-disorder*”, “*quality-of-life*” e “*critical illness*”. Recientemente, los términos más utilizados son “*impact”, “unit”* e *“rehabilitation*”, lo que refleja un enfoque centrado en los resultados tras el alta, con énfasis en la recuperación de los sobrevivientes de enfermedades críticas.

La Figura 2 muestra los términos más buscados, así como sus coocurrencias, con una frecuencia de ≥ 5 coocurrencias, lo que proporciona una mayor comprensión del panorama de la investigación.

El mapa de la red ilustra la estructura altamente interconectada y multidisciplinaria de la investigación centrada en el “*post intensive care syndrome*”, que emerge como un elemento central de la red, conectando con los principales temas de la literatura. Entre ellos, se destaca el término “*critical illness*”, el foco más sólido del campo, con 96 ocurrencias y una fuerza de enlace total de 813.

Hacen parte de esta red otros clústeres temáticos (salud mental, *delirium*/manejo clínico, rehabilitación y función cognitiva), demostrando la complejidad del campo de estudio. El *cluster* rojo se centra en las repercusiones emocionales y los determinantes psicosociales asociados a las PICS. Los estudios aquí reunidos enfatizan la alta carga de morbilidad psicológica. La relevancia del contexto social se evidencia por la presencia del término “*caregivers*”, que aparece 5 veces, con una fuerza de enlace total de 41. Este *cluster* tiene una fuerte sinergia con el *cluster* morado, lo que subraya la importancia de integrar el apoyo familiar y la dinámica de los cuidadores en los enfoques de *“follow-up”*.

**Figura 2:** Análisis de la coocurrencia de términos de búsqueda Aveiro, Portugal, 2024

El *cluster verde* reúne temas relacionados a la fase aguda de la enfermedad crítica. El “*delirium”* se destaca como el tema clínico más prominente, con 21 ocurrencias y 175 de fuerza de enlace total, lo que refleja su papel central como marcador de gravedad, complicación frecuente y predictor de resultados físicos, cognitivos y psicológicos en el período posterior al alta.

El *clúster* azul se centra en las dimensiones físicas de la recuperación. Los conceptos de “*rehabilitation*” y “*recovery*” estructuran este grupo, demostrando la creciente atención que se presta a las intervenciones de rehabilitación física y a la recuperación funcional tras la estancia en la UCI.

Finalmente, el *clúster* amarillo abarca los efectos cognitivos persistentes tras una enfermedad crítica, incluyendo déficits de memoria, atención y funciones ejecutivas. La “*quality of life*” surge como un punto central, vinculando las secuelas cognitivas y funcionales con los resultados generales de los sobrevivientes.

Los grupos de términos indican una transición de la investigación diagnóstica para la gestión integrada y personalizada, con un enfoque creciente en los mecanismos biológicos y la experiencia del paciente.

### Revistas

El número de artículos publicados en revistas especializadas es un indicador de la relevancia del tema dentro de la comunidad científica. La Tabla 1 presenta las revistas que publicaron más artículos en el área de SPCI, según el h-index y el número total de citas.

**Tabla 1:** Análisis de publicaciones: índice h y citas. Aveiro, Portugal, 2024

Revistas	h-index*	g-index^†^	*TC* ^*‡*^	*NP* ^*§*^	*PY-start* ^*||*^
*Critical Care*	7	10	543	10	2016
*BMJ Open*	6	8	201	8	2017
*Critical Care Medicin*e	5	8	800	8	2014
*Annals Of The American Thoracic Society*	3	4	160	4	2016
*Critical Care Explorations*	3	4	36	4	2022
*Current Opinion In Critical Care*	3	5	79	5	2012
*Intensive And Critical Care Nursing*	3	4	32	4	2020
*Journal Of Intensive Care*	3	3	52	3	2023
*Acute Medicine & Surgery*	2	2	8	2	2024
*Australian Critical Care*	2	3	24	3	2022
*Critical Care Nurs*e	2	2	8	2	2022
*Journal Of Advanced Nursin*g	2	2	8	2	2021
*Journal Of Clinical Medicine*	2	3	15	3	2021
*Journal Of Critical Care*	2	3	184	3	2015
Medicina Intensiva	2	2	17	2	2019

*h-index = Métrica que proporciona una visión equilibrada de la productividad y el impacto del autor; ^†^g-index = Índice que amplifica las citas de los artículos más citados para destacar las distinciones entre investigadores; ^‡^ *TC* = Total de citas; ^§^ *NP* = Número de publicaciones; ^||^ *PY-start =* Primera publicación

La revista con más publicaciones y mayor relevancia es la revista “*Critical Care”* que publicó un total de 10 artículos entre 2016 y 2024, con 543 citas, y que tiene un h-index de 7 y un g-index de 10.

Esta lista incluye ocho revistas con su primera publicación sobre el tema en análisis a partir del año 2020, evidenciando una creciente consolidación del tema como un campo relevante de investigación.

Además, es importante mencionar que en esta lista se incluyen tres revistas de enfermería: “*Intensive And Critical Care Nursing”*, *“Critical Care Nurse”* y *“Journal of Advanced Nursing*”, que proporcionan información sobre el impacto y la consistencia de las publicaciones de estas revistas en el campo de la enfermería y los cuidados intensivos.

### Análisis de autores y citas

La Tabla 2 resume a los autores con respecto a las contribuciones realizadas en el ámbito de la investigación sobre SPCI.

**Tabla 2:** Análisis de citas - evaluación del autor. Aveiro, Portugal, 2024

Autores		h-index*	g-index^†^	*TC* ^*‡*^	NP^§^	PY start^||^
Needham D.M.		8	12	808	12	2014
Sevin C.M.		7	9	501	9	2018
Hopkins R.O.		6	7	668	7	2012
Jackson J.C.		6	7	765	7	2018
Akhlaghi N.		4	4	150	4	2019
Bose S.		4	4	150	4	2019
Brown S.M.		4	4	150	4	2019
Dinglas V.D.		4	5	150	5	2019
Ely E.W.		4	4	434	4	2018

* *h-index =* Métrica que proporciona una visión equilibrada de la productividad y el impacto del autor*;* ^*†*^ g-index = Índice que amplifica las citas de los artículos más citados para destacar las distinciones entre investigadores*;* ^*‡*^ TC = Citas totales*;* ^*§*^ NP = Número de publicaciones; ^||^ *PY_start* = Primera publicación

Evaluado en términos de número de artículos publicados, h-index, g-index g y número total de citas obtenidas, se destaca Needham, con 12 publicaciones y con el mayor número de citas: 808, quien, junto con Hopkins, es uno de los autores que publica desde hace más tiempo sobre la temática.

Con publicaciones más recientes, desde 2018, se destacan Sevin, con nueve publicaciones y 501 citas, es decir, el impacto de sus artículos está bien representado por el número de citas de sus artículos más citados; y Jackson, que presenta 7 artículos con un total de 765 citas y un h-index de 6. Este autor cuenta con una base sólida de trabajos bien citados, con un impacto significativo.

La Figura 3 presenta una lista de los artículos más citados, identificando los autores, el año de publicación, el título del artículo y el número de citas.

De los artículos más citados, se destacan “*Co-occurrence of Post-Intensive Care Syndrome Problems Among 406 Survivors of Critical Illness*” y “*Anxiety, Depression and Post Traumatic Stress Disorder after Critical Illness: a UK wide prospective cohort study*”, ambos de 2018.

**Figura 3:** Análisis de citas. Aveiro, Portugal, 2024

Autor, año	Título	Citas
Marra, et al., 2018	*Co-occurrence of Post-Intensive Care Syndrome Problems Among 406 Survivors of Critical Illness*	287
Elliott, et al., 2014	*Exploring the Scope of Post-Intensive Care Syndrome Therapy and Care: Engagement of Non-Critical Care Providers and Survivors in a Second Stakeholders Meeting*	281
Hatch, et al., 2018	*Anxiety, Depression and Post Traumatic Stress Disorder after critical illness: a UK wide prospective cohort study*	281
Mikkelsen, et al., 2020	*Society of Critical Care Medicine’s International Consensus Conference on Prediction and Identification of Long-Term Impairments After Critical Illness*	202
Fuke, et al., 2018	*Early rehabilitation to prevent postintensive care syndrome in patients with critical illness: a systematic review and meta-analysis*	130
Sevin, et al., 2018	*Comprehensive Care of ICU Survivors: Development and Implementation of an ICU Recovery Center*	126
Brown, et al., 2019	*Approaches to Addressing Post-Intensive Care Syndrome among Intensive Care Unit Survivor*	126
Gayat, et al., 2018	*Determinants of long-term outcome in ICU survivors: results from the FROG-ICU study*	124
Schofield-Robinson, et al., 2018	*Follow-up services for improving long-term outcomes in intensive care unit (ICU) survivors (Review)*	121
Jensen, et al., 2015	*Impact of follow-up consultations for ICU survivors on post-ICU syndrome: a systematic review and meta-analysis*	110
Major, et al., 2016	*Surviving critical illness: what is next? An expert consensus statement on physical rehabilitation after hospital discharge*	80

El artículo^25^ *“Exploring the Scope of Post-Intensive Care Syndrome Therapy and Care: Engagement of Non-Critical Care Providers and Survivors in a Second Stakeholders Meeting”*, con 281 citas, contribuyó a aumentar la concienciación y la educación sobre SPCI.

Con más de 100 citas y publicado en 2019, se encontró el artículo^26^ ^)^ “*Approaches to Addressing Post-Intensive Care Syndrome among Intensive Care Unit Survivor”.*

### Distribución de la producción científica

La distribución de la producción científica en SPCI, considerando tanto las colaboraciones internacionales como las publicaciones de autoría nacional, reveló que los Estados Unidos lidera significativamente tanto en el número absoluto de publicaciones como en colaboraciones internacionales, lo que evidencia su centralidad en la investigación sobre este tema.

Los Países Bajos y Japón aparecen a continuación, demostrando un fuerte compromiso con la producción científica y destacándose por su activa red de colaboración global. Por su parte, China, a pesar de presentar un volumen considerable de publicaciones, no estableció colaboraciones internacionales significativas dentro de la muestra analizada.

Los resultados sugieren que la producción científica sobre SPCI está concentrada en determinados polos académicos, siendo impulsada por países que demuestran mayor inversión en la investigación sobre cuidados tras el alta de la UCI, incluida la rehabilitación.

## Discusión

El análisis bibliométrico, a pesar de haberse realizado únicamente en Web of Science, permitió responder a las preguntas de investigación formuladas y ofrecer un panorama amplio sobre la evolución de la investigación relacionada con SPCI, así como verificar una tendencia creciente en artículos de investigación originales y revisiones sobre SPCI. No obstante, esta limitación, junto con el número reducido de artículos, puede introducir un sesgo de selección que reduce la generalización de los resultados.

El análisis de los términos más utilizados en la investigación permite ofrecer sugerencias valiosas sobre el panorama de la investigación^20^ ^,^ ^27^. Es posible observar la aparición de términos emergentes como *“impact”* y *“rehabilitation”*. Estos términos reflejan la transición de la investigación inicial, que enfatizaba la caracterización del síndrome, hacia un abordaje más aplicado, orientado a la rehabilitación y a la calidad de vida, que es afectada significativamente tras la hospitalización en la UCI^8^ ^,^ ^19^. Los elementos de los clústeres confirman la complejidad multidimensional del Síndrome Post-Cuidados Intensivos, amplían la comprensión con nuevas dimensiones y tecnologías, y cuestionan el modelo de seguimiento Post-Hospitalario en Cuidados Intensivos, así como la heterogeneidad metodológica.

La evolución de la investigación a lo largo de los años demostró un aumento del interés por temas como el trastorno de estrés postraumático, la calidad de vida y el impacto de la enfermedad crítica, destacando la necesidad de enfoques holísticos e intervenciones multidisciplinares que faciliten la adaptación del paciente a su nueva situación de salud. El conocimiento descriptivo ha madurado; sin embargo, la investigación interventiva, predictiva y traslacional sigue estando poco desarrollada.

La bibliometría reveló que la investigación sobre SPCI se concentra en revistas científicas de gran relevancia en el área de los cuidados críticos y la medicina intensiva, destacándose las revistas: *Critical Care* ^22^ *, BMJ Open* e *Critical Care Medicine* ^23^, que presentaron los mayores índices de productividad y citas. La presencia de tres revistas específicas de enfermería en la lista de las más relevantes demuestra la creciente importancia que los enfermeros atribuyen a la detección, el tratamiento y la rehabilitación de los supervivientes de la UCI.

Los enfermeros desempeñan un papel fundamental en la minimización de SPCI a través de intervenciones centradas en la movilización precoz, el manejo del delirium, el apoyo familiar y la planificación del alta^28^. Asimismo, en el seguimiento de los supervivientes y sus familias, las intervenciones de enfermería tienen como objetivo activar estrategias eficaces de afrontamiento, promover el autocuidado, recuperar la autonomía del paciente y prevenir la fatiga asociada al rol del cuidador^29^. El seguimiento de los pacientes tras el ingreso en la UCI representa un área emergente en el ámbito de los cuidados de salud, cuyo objetivo es identificar y abordar las consecuencias a largo plazo de la permanencia en la UCI de forma más eficaz, en comparación con los cuidados estándar^30^. No obstante, los datos disponibles son limitados en cuanto a las intervenciones eficaces que deben implementarse y evidencian la falta de estandarización de un modelo de seguimiento de los pacientes tras una enfermedad crítica^30^, así como lagunas en la implementación de enfoques integrados y longitudinales.

Se identificó una alta prevalencia de deterioro cognitivo, ansiedad, depresión, debilidad física, cambios en el estilo de vida y problemas relacionados con la medicación, que justifican la intervención en los pacientes tras la hospitalización en cuidados intensivos^31^. La SPCI está asociada con una disminución de la calidad de vida de los pacientes tras el alta de la UCI^9^. El concepto del “yo alterado” hace referencia a la transformación del “*self*”, reflejando las nuevas limitaciones físicas experimentadas, así como la manera en que la movilización de las características intrínsecas del individuo facilita la adaptación a su nueva identidad^32^.

El seguimiento de los pacientes puede ayudar a crear la narrativa de su enfermedad y recuperar un sentido de coherencia, sin embargo se necesitan ensayos clínicos aleatorios para estandarizar las intervenciones y las medidas de resultados^33^.

Los autores^5^ ^)^ recomiendan evaluaciones sistematizadas y sugieren herramientas de cribado para evaluar cada uno de los componentes del SPCI, destacando la importancia de priorizar a los pacientes de alto riesgo y derivarlos a servicios y/o evaluaciones más detalladas. Es necesaria una evaluación sistemática y frecuente del SPCI a lo largo de todo el proceso asistencial^25^.

Es importante identificar los factores de riesgo para el desarrollo de SPCI, y el equipo multidisciplinario debe estar atento a los factores de riesgo modificables. Una mayor comprensión de los factores de riesgo ha mejorado la capacidad de identificar a los pacientes de alto riesgo para el cribado y la intervención^17^. Los autores^10^ ^)^ evaluaron la incidencia de SPCI en supervivientes de COVID-19 e identificaron *el delirium* y la traqueotomía como factores asociados. El equipo de enfermería desempeña un papel preponderante, tanto en la identificación precoz de los factores de riesgo como en la implementación de *bundles*, disminuyendo el riesgo de desarrollo del SPCI.

Es importante realizar estudios que examinen la implementación de recomendaciones, incluyendo su viabilidad, aceptabilidad, sostenibilidad y fidelidad, además de evaluar el impacto de dichas recomendaciones en los resultados a largo plazo para los supervivientes^5^. La investigación debe centrarse en los pacientes y en las transiciones asistenciales^26^, ya que una transición eficaz de los cuidados debe asegurar una continuidad entre el tratamiento en la UCI, en la sala de hospitalización y el apoyo continuo en la comunidad y en el domicilio.

El análisis bibliométrico reveló, además, desigualdades en la distribución de la producción científica sobre SPCI y áreas afines entre países y continentes; se destaca la restricción lingüística adoptada (portugués, inglés y español) como un aspecto metodológico relevante de la investigación. Al igual que en otros estudios^22^ ^-^ ^23^, los Estados Unidos lideran significativamente tanto en número de publicaciones como en colaboraciones internacionales, lo que puede estar relacionado con una sólida inversión en cuidados post-UCI e investigación longitudinal. Los Países Bajos y Japón también destacan como centros de investigación activos, lo que refleja el desarrollo de estrategias avanzadas de rehabilitación y la reestructuración de los cuidados intensivos.

Por otro lado, China, a pesar de contar con un volumen significativo de publicaciones, mostró una baja colaboración internacional, posiblemente debido a barreras lingüísticas o a un modelo de investigación más centrado en las realidades clínicas internas. Portugal, por su parte, muestra una participación reducida y una ausencia de colaboraciones internacionales en la muestra analizada. Esto puede atribuirse a dificultades de financiación y a la falta de redes de colaboración con grupos internacionales de cuidados intensivos, especialmente en Europa.

El síndrome SPCI continúa siendo una preocupación creciente, y existe consenso en la literatura sobre la necesidad de mejorar los mecanismos de identificación precoz y seguimiento de los supervivientes en estado crítico. Se deben realizar evaluaciones sistemáticas y frecuentes del síndrome a lo largo de todo el proceso asistencial, priorizando a los pacientes de alto riesgo para la intervención precoz^5^, e investigar su biología molecular o predisposiciones genéticas^22^.

Estudios recientes señalan la necesidad de estrategias de transición eficaces que garanticen la continuidad de la atención entre el alta de la UCI y la reincorporación a la vida cotidiana^26^. El enfermero, como coordinador de este seguimiento, es crucial debido a su experiencia en la gestión de cuidados complejos y su capacidad de articulación entre el paciente, la familia y el equipo multidisciplinario^29^.

La creciente atención prestada a SPCI destaca la importancia de ampliar las colaboraciones internacionales, acelerar la investigación en áreas relacionadas^22^ ^)^ promoviendo redes de investigación multicéntricas y garantizando que las nuevas evidencias científicas sean aplicables a diversos escenarios clínicos. El fortalecimiento de estas redes puede contribuir a un abordaje más equitativo y eficaz para el seguimiento tras el ingreso en la UCI, beneficiando no solo a los pacientes, sino también a sus familias y a los profesionales involucrados en su recuperación/rehabilitación.

La investigación sobre la SPCI presenta limitaciones, ya que se centra principalmente en los efectos clínicos a corto plazo, con escaso análisis a largo plazo y estudios multinacionales. Además, muchos estudios analizan únicamente una dimensión del síndrome, sin explorar la interacción entre sus diferentes componentes, lo que dificulta una comprensión más integral de la SPCI. Otro desafío importante es la falta de estandarización en los instrumentos de evaluación, lo que dificulta la comparación entre estudios y limita la aplicabilidad de los hallazgos científicos. Para superar estas limitaciones, es esencial fortalecer la investigación mediante alianzas internacionales, promoviendo estudios multicéntricos y longitudinales que evalúen no solo el impacto de la SPCI en el paciente, sino también en sus familiares y cuidadores

## Conclusión

Este estudio bibliométrico presenta una síntesis integral y un análisis de las tendencias y de los temas emergentes relacionados con la SPCI, con énfasis en el seguimiento de los pacientes tras el alta de la UCI. Se mantiene la preocupación por la identificación precoz de factores de riesgo asociados al desarrollo de la SPCI y por la implementación de medidas preventivas eficaces. El seguimiento tras el alta de la UCI sigue siendo fragmentado, con diversos estudios que abordan únicamente uno de los componentes de la SPCI, sin realizar una evaluación holística del paciente, en particular la integración del dominio de la espiritualidad. Resulta esencial investigar e implementar intervenciones eficaces para el tratamiento de las diversas disfunciones asociadas a la SPCI, promoviendo una atención de calidad centrada en el paciente y en su familia, que facilite la reintegración social y funcional de ambos.

No se encontró un método ideal para el seguimiento tras el alta de la UCI, ni estudios comparativos entre los diferentes métodos: consulta presencial, telefónica, clínicas especializadas o atención domiciliaria. Actualmente, existen diversas herramientas para el cribado del SPCI, y algunos consensos recientes proponen instrumentos específicos para cada componente del síndrome. Sin embargo, sigue faltando un instrumento único y validado que evalúe de forma integrada todas las dimensiones, limitando un abordaje sistematizado e integral en la práctica clínica.

En el ámbito de la enfermería, son necesarios programas estructurados de seguimiento post-UCI, con enfoque en la evaluación funcional, el apoyo emocional y la educación para la autogestión. El seguimiento telefónico y las visitas domiciliarias pueden optimizar la continuidad de los cuidados. Futuras investigaciones deberán evaluar la eficacia de estas intervenciones y su impacto en la calidad de vida y en las readmisiones hospitalarias de los pacientes.

El avance de la investigación y la implementación de estrategias basadas en la evidencia son fundamentales para mejorar los cuidados post-UCI. Para ello, son necesarios estudios futuros bien diseñados que promuevan la cooperación internacional y contribuyan al desarrollo de prácticas clínicas más integradas y sostenibles.

## Data Availability

Todos los datos generados o analizados durante este estudio están incluidos en este artículo publicado.
